# Reports of Cases Occurring in the Medical Ward of the Buffalo Hospital

**Published:** 1858-01

**Authors:** A. W. Nichols

**Affiliations:** Assistant to Chair of Clinical Medicine, University of Buffalo


ART. IV.—*Abstract of the Clinical Lectures delivered at the Buffalo Hospital of the Sisters of Charity, upon the Diseases treated in the Male Medical Ward, by* AUSTIN FLINT, M. D., *Attending Physician, during the present College Session, viz., from October 14th,* 1857, *to February* 27, 1858. Reported by A. W. NICHOLS, M. D., Assistant to Chair of Clinical Medicine, University of Buffalo.

No. II. *For November.*

*Bulbous Enlargement of the Fingers and Toes occurring in the course of Pulmo-Tuberculosis.*

During the past two years, in several cases of pulmo-tuberculosis which have been under treatment in the wards of the hospital, there has been observed to exist a singular appearance of the fingers. This has been called by writers "clubbed fingers" or "bulbous enlargement." It is usually accompanied with an incurvation of the nails which are frequently cracked transversely.

This condition of the fingers, it is well known, does not exist in the course of every case of pulmo-tuberculosis: neither is it presented alone in this disease. It has been observed in cases of aneurism of the arch of the aorta, in cyanosis, and in empyema; but it is much more frequently met with during the course of tuberculosis.

In connection with the latter disease, it is seen now and then, and heretofore has been regarded merely as a clinical curiosity. Occurring as one of the many symptoms in the clinical history of pulmo tuberculosis, Dr. Walshe in his treatise on "Diseases of the Lungs and Heart," briefly refers to this appearance as follows:

"Bulbousness of the finger-ends, curvation and transverse cracking of the finger-nails, and falling of the hair, are observed in a certain share of cases." Evidently, regarding the enlargement of the finger-ends as a casual symptom inconsistent in its appearance, and unconnected with any particular state of the pulmonary disease.

It is now nearly a year since Prof. Flint remarked, that the bulbous enlargement of the finger-ends with incurvation of the nails, would be found to exist with extensive tuberculous solidification of one lung. Up to that time, five cases had been observed, upon which the foregoing conclusion was based. Since then, opportunities have offered to verify the accuracy of the observation.

It may be interesting to record briefly the facts developed in reference to this subject, on examination of two patients now in the hospital, who are affected with tuberculosis and present clubbed fingers and incurved nails.

Oct. 2d, 1857, Dr. F. examined the chest of a patient for the first time. Physical signs denoting extensive solidification of the right lung and comparatively little of the left, were found to exist.

Dr. F. then remarked to me, that, according to the results of observations in similar instances occurring in this hospital, we should expect to find bulbous enlargement of the fingers. On examination of the hands this appearance was found to exist in a marked degree.

Oct. 17th, 1857, Dr. F. noticed a patient presenting the clubbed finger ends with incurvation of the nails; and stated, that in accordance with what had been previously noticed, this patient should have solidification from tuberculous matter, much more extensive on one side than the other.

On examination of the chest, the physical signs afforded conclusive evidence of such a condition of the left lung.

In all, *nine* cases have been observed to present the foregoing conditions coëxisting. In every case presented in the hospital during the past two years, in which there has existed very general solidification of one lung and comparatively little of the other, from tuberculous matter, the bulbous fingers have likewise been found. And in every case, during the same period, in the same institution, in which the bulbous finger-ends with incurved nails, have been observed, there has coëxisted extensive solidification of one lung and but little comparatively of the other.

Taking out of consideration those rare forms of disease in which this enlarged condition of the finger-ends is said to have been occasionally noticed, it may be stated as a rule, that *whenever there is present bulbous enlargement of the finger-ends with incurvation of the nails, there coëxists more or less extensive solidification of one lung from tuberculous disease;* the other being very slightly affected.

This rule, it should be borne in mind, has been deduced from observations made in *nine* successive cases: and, with a single exception, extended over a period of nearly two years.

Since these results have been obtained, the writer has directed his attention to the toes: and in three cases, being all which have come under his notice, in which the bulbous finger-ends and incurved nails exist in a marked degree, there has been found a similar condition of the ends of the toes and of the nails, but in a less marked degree. It would appear, therefore, that this enlargement of the extremities of the digits is not confined to the fingers, but exists both in the fingers and the toes. The same inference would apply to the incurvation of the nails.

The practical importance of this clinical discovery is not insignificant, as —at first sight—might be supposed. If the rule we have announced hold good, this peculiar condition of the fingers would be of no little aid to us in those instances in which some cause or causes prevent us from examining the chest, and more often in those cases where the physical signs might be obscure or of doubtful interpretation; and in any case, as additional testimony to the accuracy of our diagnosis.

### *Case of Tuberculosis and sub-acute Rheumatism coëxisting; Effect of Iodide of Potassium.*

This patient has been under observation for a number of years. The pulmonary disease is singularly slow in its progress. The attacks of rheumatism are pretty frequent; and are sub-acute; that is, of less intensity than the acute, but still confined to the joints. The rheumatic affection has yielded generally very readily to treatment.

The remedy which has, by repeated trials, proved the most efficacious, is the iodide of potassium. This medicine has invariably given relief, and in a very short time the rheumatic attack has disappeared. When continued for any length of time, sometimes but a few days, an eruption has been observed, on the face more especially; this is at first papular, but soon becomes vesicular and pustular. Cases in which tuberculosis and rheumatism coëxist, are comparatively rare. Patients affected with the former disease are, as a general rule, exempt from attacks of the latter disease; and *vice versa.* It is interesting to observe in the foregoing case, the slow progress of the pulmonary affection, and the readiness with which the rheumatism yields to treatment.

### *Gastralgia and Enteralgia.*

Two cases of neuralgia affecting different portions of the alimentary cana have been under treatment in the ward. In one instance, the stomach is the seat of pain; and in the other, the duodenum probably.

When neuralgia affects the digestive apparatus, it is said to be persistent; and the cure of it difficult. Usually, there is found more or less tenderness over the spine; but, in both these cases, this was very slight indeed, and observed for a short time only.

These patients complain of sharp and lancinating pain referred to the regions of the stomach and of the umbilicus respectively. The pain is not continuous, but in both cases has been paroxysmal. The patients are attacked suddenly and in the night time, being awakened from sleep by sharp pain, which gradually increases and becomes cutting or lancinating. After continuing for six or eight hours, the pain slowly subsides, and merely a sense of soreness remains in the respective regions of the stomach and of the umbilicus.

The disease in both instances has proved singularly obstinate to treatment; the pain has not even showed a disposition to abate. Morphia, when administered before the paroxysm commenced or during its continuance, has deferred the attack of pain or has produced great relief. But, the paroxysms have invariably recurred. A faithful trial of quinia, and of arsenic, of bismuth, and of iodide of potassium, has been made. But, the results have been unsatisfactory. Under the use of the sub-nitrate of bismuth in large doses, from ʒss to ʒj, three times daily, there seemed to be in each case, for a short time, a decided improvement. But, this medicine soon appeared to be of very little, if any benefit.

